# Resistin directly inhibits bacterial killing in neutrophils

**DOI:** 10.1186/s40635-019-0257-y

**Published:** 2019-05-30

**Authors:** Lauren Miller, Kai Singbartl, Zissis C. Chroneos, Victor Ruiz-Velasco, Charles H. Lang, Anthony Bonavia

**Affiliations:** 1 Department of Anesthesiology and Perioperative Medicine, Penn State Milton S. Hershey Medical Center, 500 University Dr, Mail Code H-187, Hershey, PA 17033 USA; 2 Department of Critical Care Medicine, Mayo Clinic, Phoenix, AZ USA; 3 Department of Pediatrics, Penn State Milton S. Hershey Medical Center, Hershey, PA USA; 4 Department of Microbiology and Immunology, Penn State Milton S. Hershey Medical Center, Hershey, PA USA; 5 Department of Cellular and Molecular Physiology, Penn State Milton S. Hershey Medical Center, Hershey, PA USA; 6 Department of Surgery, Penn State Milton S. Hershey Medical Center, Hershey, PA USA

**Keywords:** Resistin, Sepsis, Neutrophils, Macrophages, Gram negative, Gram positive, Reactive oxygen species

## Abstract

**Background:**

Sepsis-induced immunosuppression is a key factor contributing to the morbidity and mortality of critically ill patients, and polymorphonuclear neutrophil dysfunction is believed to be a hallmark of this immunosuppression. Circulating myeloid cells produce the cytokine resistin (RETN), which has been associated with poor outcomes in sepsis/septic shock and can directly inhibit neutrophil function. We previously demonstrated that resistin caused a dose-dependent impairment in neutrophil migration, reactive oxygen species production, and bacterial clearance in neutrophil cell lines. However, the relative antimicrobial responses of other innate immune cells to Gram-positive and Gram-negative infections in the presence of elevated levels of resistin have not been evaluated. We hypothesized that resistin directly contributes to sepsis-induced immunosuppression by selectively targeting the neutrophil component of the innate cellular immune system. Thus, the goal of the present study was to compare the effect of resistin on bacterial killing using monocultures or co-cultures of monocyte and neutrophil cell lines, as well as to extend our findings to primary immune cells.

**Results:**

Our results indicate that human resistin impairs the ability of neutrophils to kill the Gram-negative bacterium *Pseudomonas aeruginosa* and the Gram-positive bacterium *Staphylococcus aureus*. In contrast, with the exception of macrophages incubated with *P. aeruginosa*, resistin did not affect the ability of macrophages or monocytes to kill either Gram-positive or Gram-negative organisms. Furthermore, co-incubation of neutrophils with increasing proportions of monocytes did not enhance bacterial killing. Resistin blocked bactericidal activity through partial reduction of F-actin polymerization and suppression of the oxidative burst in neutrophils.

**Conclusions:**

Our studies indicate that resistin selectively impairs neutrophil bacterial killing. These findings further support the notion that resistin can mimic cell type-dependent immunosuppressive effects. This is consistent with its putative role in the pathogenesis of bacterial sepsis.

## Background

Sepsis-induced morbidity and mortality is increasingly being attributed to global immune dysfunction, including immunosuppression [1, 2]. Cytokines, key regulators of a host’s immune response to infection, are crucial both for mounting an appropriate response to infection and for its timely abatement at the resolution of the infection [3]. In essence, these soluble mediators coordinate the movement and responses of immune cells during inflammatory states, making them likely mediators in sepsis-induced immunosuppression.

Our group and others have shown that resistin (RETN), a recently discovered inflammatory cytokine, is significantly upregulated in septic shock and sepsis-associated acute kidney injury (AKI) [4–12]. There are two circulating forms of human resistin: RETN (produced by human immune cells including macrophages, monocytes, and neutrophils) and RETNLB (produced predominantly by human intestinal goblet cells) [13]. While hyperresistinemia (> 20 ng/mL serum RETN concentration) predicts a greater disease severity and a worse prognosis in sepsis [5, 6, 8], the precise receptor [13], signaling mechanism, and effects of this cytokine on immune cells remain unknown.

Neutrophils and monocytes provide the first line of defense against infection, and their function may be adversely affected by hyperresistinemia. After migrating into tissues, monocytes differentiate into tissue macrophages to eradicate invading pathogens. Macrophages may also be adversely affected by exposure to elevated resistin concentrations. Neutrophils, monocytes, and macrophages act in a coordinated fashion to phagocytose and destroy invading organisms as well as to trigger the development of an adaptive immune response [14].

We have previously shown that resistin, at concentrations commonly found in septic human patients, impairs neutrophil migration in a dose-dependent manner by impairing F-actin polymerization [9]. While it does not affect neutrophil phagocytosis [4, 15], resistin also blocks the bacterial killing of internalized bacteria, possibly by inhibiting the production of reactive oxygen species [15]. However, data regarding the relative responses of other myeloid cells to resistin remains sparse. We hypothesized that unlike macrophages and monocytes, neutrophils are directly and selectively inhibited by resistin both in the context of Gram-positive and Gram-negative infections.

We tested this hypothesis by exposing innate immune cells to two common and often deadly nosocomial agents whose pathogenesis varies significantly: *Pseudomonas aeruginosa*, a Gram-negative organism, and *Staphylococcus aureus*, a Gram-positive organism. We utilized neutrophil, monocyte, and macrophage cell lines in order to ensure standardization and reproducibility. We subsequently validated our results in primary cells isolated from healthy human volunteers. In order to model the physiological environment of the cytokine milieu in vivo, we co-incubated monocytes and neutrophils in the presence or absence of resistin. We assessed whether bacterial killing capacity was altered based on the ability of these myeloid cells to interact together in the presence of resistin and infection. Our findings demonstrate that resistin selectively and profoundly impairs neutrophil bacterial killing, consistent with the premise that this cytokine alone can reproduce the immunosuppressed cellular phenotype characteristic of sepsis and septic shock.

## Materials and methods

### Reagents

All reagents, including all-trans retinoic acid (R2625), PMA (P1585), Histopaque 1119 and 1077, and cytochalasin B (C6762), were obtained from Sigma-Aldrich (St. Louis, MO, USA) unless stated otherwise. In the present study, we compared neutrophil bacterial killing to that of monocytes and macrophages by using concentrations of human resistin (100 ng/mL) equivalent to those found in septic shock, and approximately five times higher than that found in control subjects [9, 15]. As a positive control, some cells were exposed to cytochalasin B, which is a cell-permeable mycotoxin that inhibits F-actin polymerization and critical cellular functions.

### Neutrophilic differentiation of NB4 acute promyelocytic leukemia cells

NB4^PMN^ cells were used to study the effect of RETN on neutrophil function. The NB4^PMN^ cell line offers the advantage of standardization and reproducibility when compared with primary neutrophils [9, 15]. Neutrophilic differentiation was achieved as previously described [9]. Briefly, NB4 cells were treated with 1 μM all-trans retinoic acid (ATRA) for 6 days in Roswell Park Memorial Institute medium (RPMI 1640) (VWR, Radnor, PA) with l-glutamine containing 10% fetal bovine serum (FBS) (Atlanta Biologicals; Flowery Branch, GA). Successful neutrophil differentiation was assessed by flow cytometry to monitor expression of CD11b, CD35, and CD71 surface markers (BD Biosciences, San Jose, CA). In some experiments, NB4^PMN^ cells were incubated for 1 h in human control serum (Atlanta Biologicals) containing 100 ng/mL of recombinant human resistin (R&D Systems, Minneapolis, MN) to replicate concentrations measured in patients with sepsis-induced AKI [15].

### THP-1 monocyte culture

THP-1 monocytes (ATCC, Manassas, VA) were grown in RPMI 1640 medium with l-glutamine containing 10% FBS. THP-1 monocytes were maintained at a density of between 10^5^ and 10^6^ cells/mL between experiments, according to published methods [16].

### Macrophage differentiation of THP-1^mac^ cells

THP-1 monocytes were differentiated into macrophages (THP-1^mac^) by incubation with 100 nM phorbol 12-myristate 13-acetate (PMA) for 72 h [17]. Differentiated cells were incubated in fresh RPMI media for 24 h before initiation of experimental studies. Prior to experimentation, successful macrophage differentiation was confirmed by expression of CD14, CD16, and CD71 surface markers (BD Biosciences) with flow cytometry.

### Primary neutrophils from human subjects

Primary neutrophils and monocytes were isolated from peripheral whole blood obtained from human subjects. The Penn State Hershey Medical Center Institutional Review Board approved the study protocol prior to initiation (PROTOCOL# 10357, approved November 2018). After informed consent, blood was drawn from healthy adult, non-pregnant subjects in K2 EDTA vacutainers (BD Biosciences, San Jose, CA). All experiments were performed within 1 h of blood sample collection. Neutrophils and monocytes were separated by Histopaque density gradient centrifugation according to the manufacturer’s instructions. Thereafter, 3 mL of Histopaque 1119 was added to a conical tube, followed by an additional 3 mL layer of Histopaque 1077. Afterwards, up to 6 mL of whole blood was added to the upper gradient of the conical tube. Samples were centrifuged at 700×*g* for 30 min at room temperature. The serum layer was harvested and warmed to 37 °C for later use. Primary neutrophils were harvested from the granulocyte layer and washed twice in PBS. To lyse remaining red blood cells, 1 mL of distilled water was added to cell pellets for 1 min, followed by PBS. Cell viability and counts were assessed using flow cytometry. Primary neutrophils or monocytes were exposed to recombinant human resistin (100 ng/mL) spiked donor-matched serum for 1 h at 37 °C, 5% carbon dioxide prior to functional assays. Donor-matched serum was also used for bacterial opsonization needed for effective bacteria killing.

### Bacterial clearance assay

*Pseudomonas aeruginosa* strain UI-18 (PA-7) (ATCC) and *Staphylococcus aureus* strain Aureus Rosenbach (ATCC) were used to determine the bacterial clearance capacity of THP-1, THP-1^mac^, NB4^PMN^ cells, and primary neutrophils, as previously described [18]. Briefly, bacteria were suspended in PBS at a concentration of 10^7^ cells/mL and opsonized with 10% human serum by using end-over-end rotation at 37 °C for 20 min [15]. Cytochalasin B (10 μM) served as positive control for actin cytoskeleton inhibition. Afterwards, 10^7^ immune cells (with or without cytochalasin B) were incubated with opsonized bacteria for 30 min with end-over-end rotation at 37 °C. One milliliter of this mixture was removed, added to ice-cold PBS, and then centrifuged at 100×*g*, for 5 min. One millilter of PBS with 0.05% saponin was then added to the cell pellet of the centrifuged mixture. Cells were disrupted with a glass homogenizer in order to release viable bacteria from the host cell cytoplasm (intracellular bacteria), as previously described [18]. The contents were then plated onto growth medium. Bacterial culture plates were incubated at 37 °C overnight, and the colony number on each plate was counted the following morning in a blinded manner.

### Quantification of reactive oxygen species generation in neutrophils

Reactive oxygen species (ROS) generation was measured by using CellROX Deep Red Reagent probe (Invitrogen, Carlsbad, CA) as previously described [15]. Briefly, 10^6^ NB4^PMN^ cells/mL were washed with 1% BSA in PBS and stimulated without or with 100 ng/mL (162 nM) PMA for 10 min at 37 °C. Ice-cold PBS was added to terminate the reaction, and diluted CellROX probe (7.5 μM) was added and incubated for 10 min at 37 °C. Samples were fixed in 4% formaldehyde in PBS (Boston BioProducts, Boston, MA) and analyzed by flow cytometry.

### Immune cell co-incubation experiments

*THP-1 cells* (monocytes) and *NB4*^*PMN*^ were incubated for 1 h in human serum in the presence and absence of 100 ng/mL recombinant human resistin (R&D Systems). Following the incubation period, cells were washed, counted, and mixed at different neutrophil-to-monocyte ratios for experimentation. The physiologic proportion of neutrophils to monocytes in human blood (which ranges from 3:1 to 7:1—we used 3:1 for convenience) was simulated, and this was then compared to a mixture of 1:1 neutrophils to monocytes. The total number of immune cells (monocytes and neutrophils) in each experimental sample was kept equal. *S. aureus* or *P. aeruginosa* were then added to the serum in the transwell plates and incubated at 37 °C for 1 h. The contents of each treatment were collected and subjected to the bacterial clearance assay.

### Experimental controls

*S. aureus* and *P. aeruginosa* were exposed to resistin (100 ng/mL) in order to determine the antimicrobial effect of resistin alone. Further, NB4^PMN^ and THP-1 cells were exposed to 100 ng/mL resistin in order to ensure that resistin exposure alone does not cause cell death. Cellular apoptosis was assessed by Annexin V staining (Thermo Fisher Scientific, Waltham, MA) followed by fluorescence-activated cell sorting (FACS) analysis.

Data analysis was performed using Prism 8 (GraphPad Software, San Diego, CA). All data are presented as median (interquartile range). Statistical analysis included, where indicated, test for normality (Shapiro-Wilk), paired and unpaired *t* tests, one-way analysis of variance (ANOVA) with Tukey’s multiple comparisons test, two-way ANOVA with Tukey’s multiple comparisons test, Wilcoxon matched-pairs signed rank test, and Mann-Whitney test. A *P* value less than 0.05 was considered statistically significant.

## Results

In preliminary experiments, we confirmed that resistin (100 ng/mL) alone did not have antimicrobial or proliferative effects on *P. aeruginosa* or *S. aureus* growth*.* We also found that resistin alone induced ~ 10% cellular apoptosis in neutrophils and monocytes (data not shown).

**Fig. 1:**
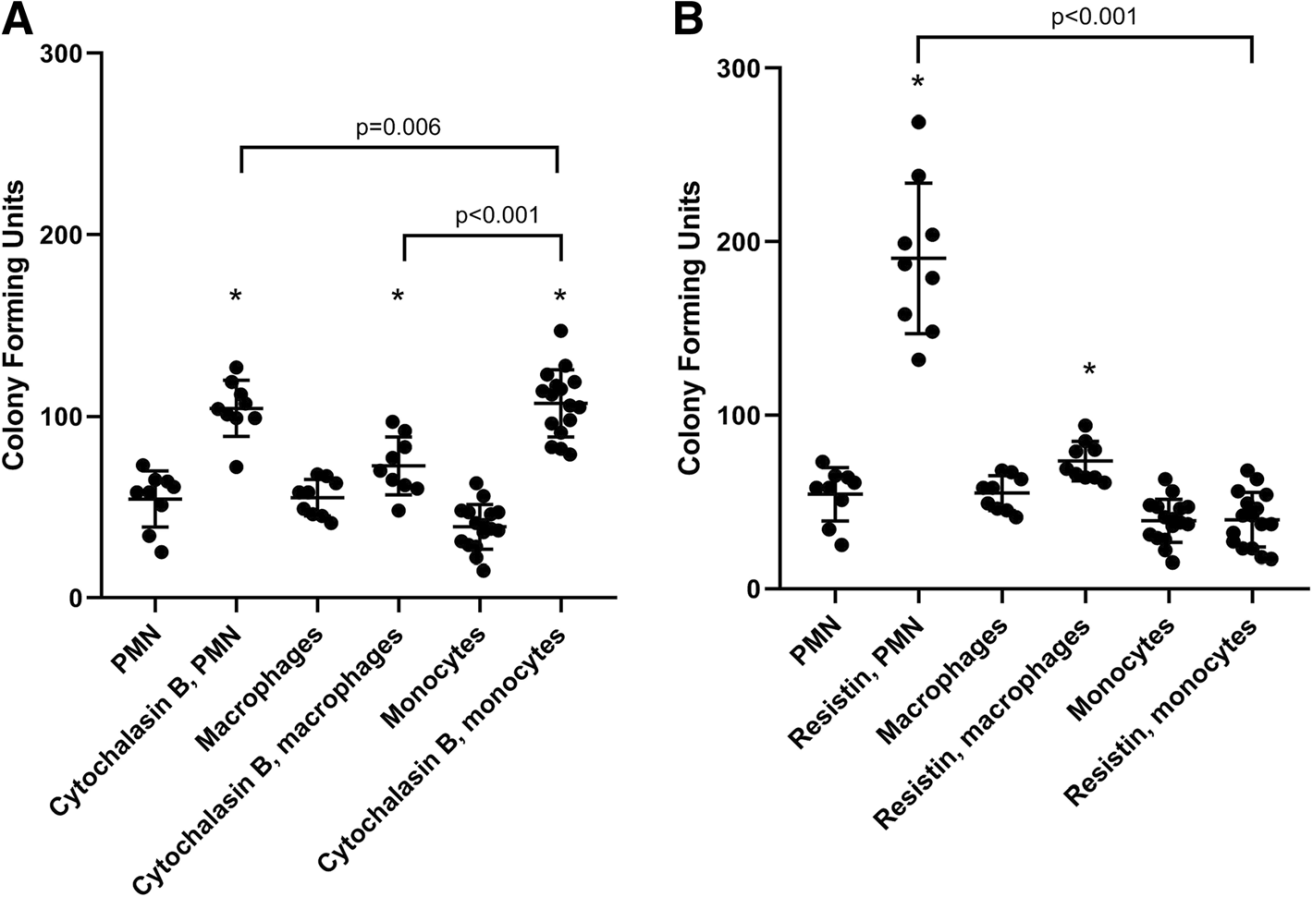
Comparison of bacterial killing of *P. aeruginosa* by PMN (neutrophils), macrophages, and monocytes in the presence of **a** cytochalasin B and **b** human resistin. Cytochalasin B significantly impaired the ability of all cell types to kill *P. aeruginosa* (*P* < 0.001 for PMN, *P* = 0.002 for macrophages, *P* < 0.001 for monocytes), underlying the importance of F-actin in the bactericidal process. Resistin significantly impaired bacterial killing of *P. aeruginosa* in neutrophils (*P* < 0.001) and macrophages (*P* < 0.001) but not in monocytes (*P* > 0.99). *n* = 18 (PMN), 9 (macrophages), and 16 (monocytes). Statistically significant differences in bacterial killing capacity between different cell types to each stimulant are denoted by *P* values on the graph. Asterisk denotes statistical significance (*P* < 0.05)

Figure 1a shows a comparison between the killing capacity of *P. aeruginosa* by neutrophils, monocytes, and macrophages in the presence of the mycotoxin, cytochalasin B. It can be observed that all immune cell types exhibited significantly impaired bacterial killing in the presence of cytochalasin, since all these cells depend on F-actin polymerization for critical cellular functions. However, there was no apparent difference in killing of *P. aeruginosa* between the three cell types. Figure 1b illustrates that resistin exposure impaired neutrophil- and macrophage-mediated bacterial killing, although the inhibitory action was more pronounced with neutrophils (*P* < 0.001). On the other hand, resistin did not significantly (*P* > 0.99) affect bacterial killing of monocytes.

**Fig. 2:**
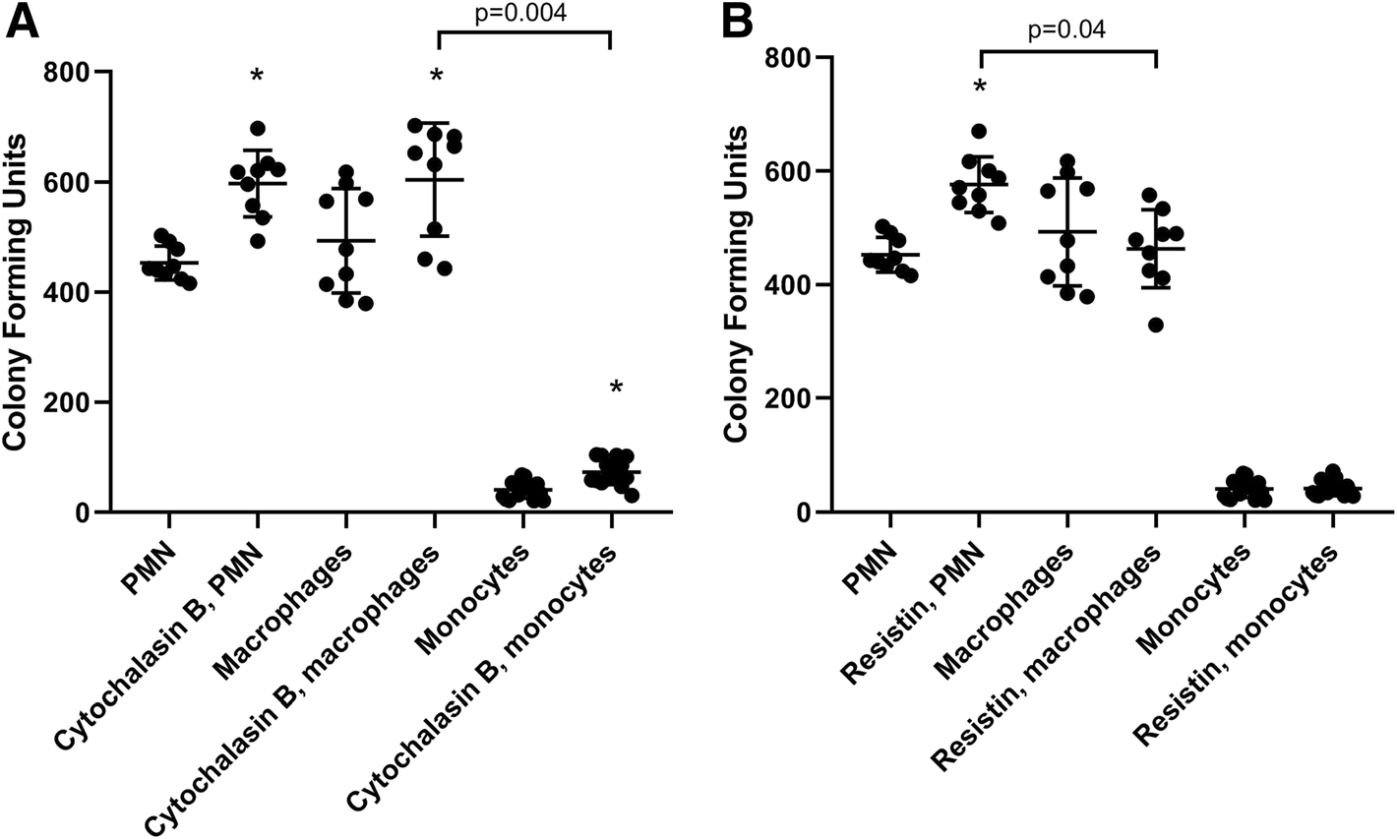
Comparison of bacterial killing of *S. aureus* by PMN (neutrophils), macrophages, and monocytes in the presence of **a** cytochalasin B and **b** human resistin. Cytochalasin B significantly impaired the ability of all cell types to kill *S. aureus* (*P* < 0.001 in PMN, macrophages, and monocytes). Human resistin significantly impaired bacterial killing of *S. aureus* in neutrophils (*P* < 0.001) but not in monocytes (*P* = 0.34) or macrophages (*P* = 0.224). *n* = 9 (PMN), 9 (macrophages), and 16 (monocytes). Statistically significant differences in bacterial killing capacity between different cell types to each stimulant are denoted by *P* values on the graph. Asterisk denotes statistical significance (*P* < 0.05)

In the next set of experiments, neutrophil, monocyte, and macrophage cells were exposed to *S. aureus* in order to assess the response of these different immune cell types to Gram-positive infection in the presence of resistin. Figure 2a shows that monocytes exhibited a greater ability to kill bacteria than either neutrophils or macrophages. Moreover, impaired F-actin polymerization via cytochalasin exposure inhibited all three immune cell types from *S. aureus* killing (Fig. 2a). On the other hand, resistin exposure inhibited bacterial killing of neutrophils (*P* < 0.001; Fig. 2b) but it did not effect on the ability of monocytes or macrophages to kill *S. aureus*.

**Fig. 3:**
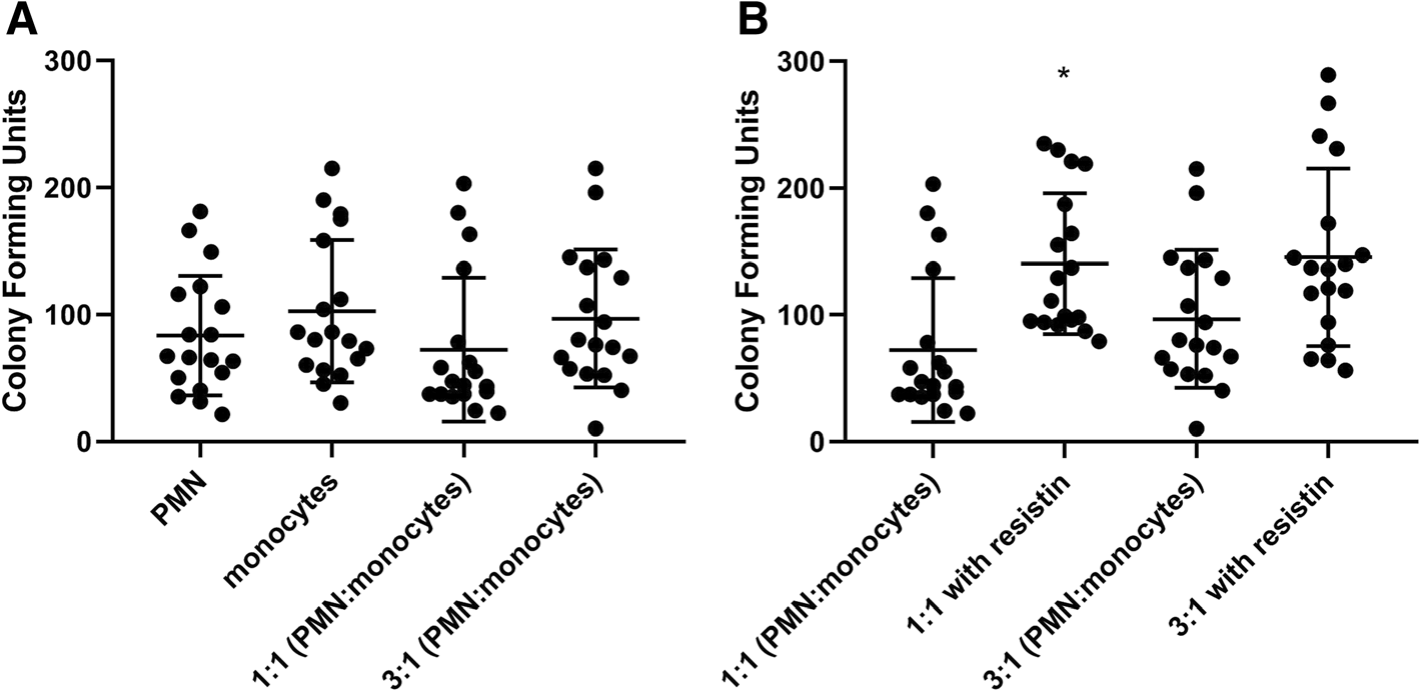
Effect of co-incubation of PMN (neutrophils) and monocytes on bacterial killing of *P. aeruginosa* with different ratios of PMN to monocytes in the **a** absence of resistin and **b** presence of resistin. Direct co-incubation was achieved by mixing PMN and monocytes in 1:1 ratio and 3:1 ratio (total cell counts kept the same in each condition). Co-incubation with increasing proportions of monocytes did not improve killing of *P. aeruginosa*. *n* = 18 for all experiments. Asterisk denotes statistical significance (*P* < 0.05)

**Fig. 4:**
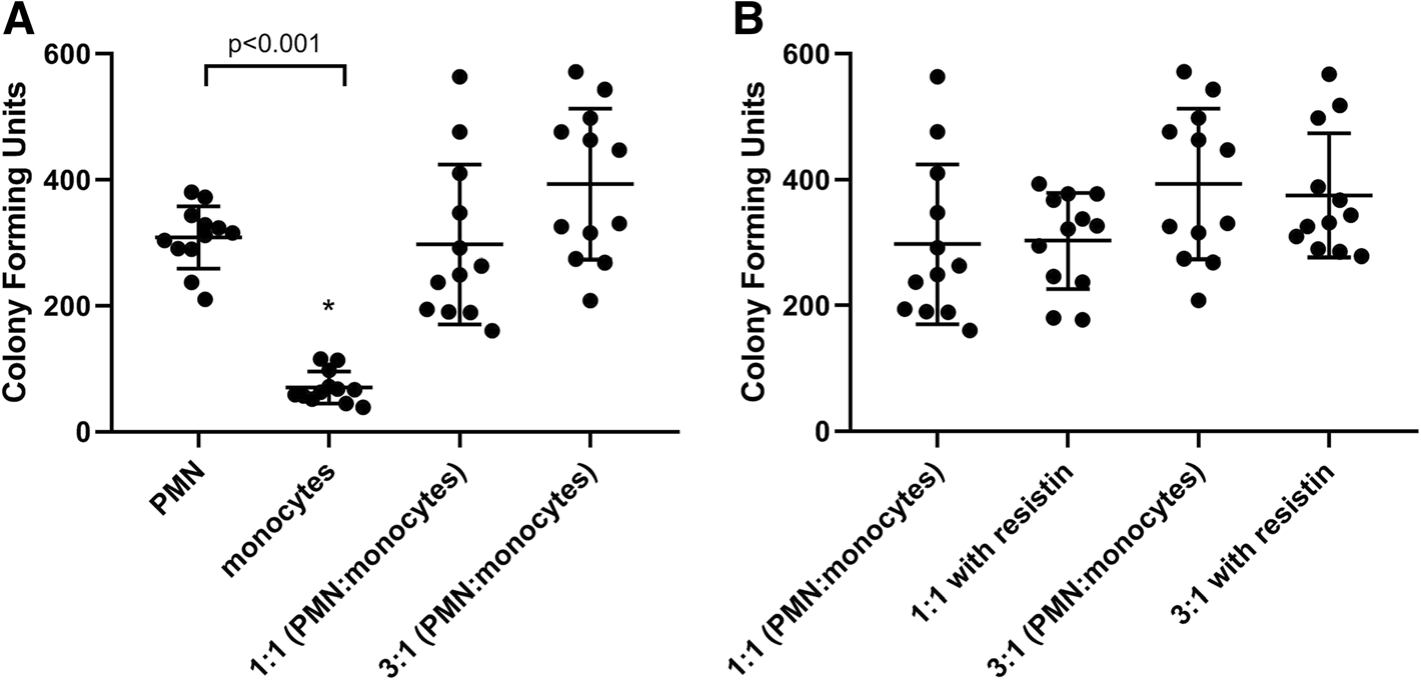
Effect of co-incubation of PMN (neutrophils) and monocytes on bacterial killing of *S. aureus* with different ratios of PMN to monocytes in the **a** absence of resistin and **b** presence of resistin. Direct co-incubation was achieved by mixing PMN and monocytes in 1:1 ratio and 3:1 ratio (total cell counts kept the same in each condition). Co-incubation with increasing proportions of monocytes did not improve killing of *S. aureus*, neither did it cause any change in bacterial killing in the presence of resistin. *n* = 12 for all experiments. Asterisk denotes statistical significance (*P* < 0.05)

Studies have shown that monocytes produce cytokines which may alter the response of leukocytes to bacterial cell products such as lipopolysaccharides (LPS) [19]. Therefore, to investigate how different types of innate immune cells interact in vitro, we co-incubated neutrophils and monocytes together at different cellular ratios. Cells were allowed to come into contact, in order to facilitate the interaction between both cell types via released mediators. The data shown in Figs. 3 and 4 depict that there was a lack of neutrophil-monocyte synergism in regard to killing the Gram-negative or Gram-positive bacteria in our in vitro model of infection. When compared with the baseline ability of neutrophils to kill *P. aeruginosa*, there was no difference in the bacterial killing of neutrophils mixed with monocytes at a 1:1 ratio or at a 3:1 ratio (*P* = 0.96 and *P* = 0.93, respectively; Fig. 3). Similarly, there was no effect of the neutrophil to monocyte ratio on the killing of *S. aureus*, compared to neutrophils alone (*P* > 0.99 and *P* = 0.32 for 1:1 and 3:1 ratios of cells, respectively). In a separate set of experiments, both neutrophils and monocytes were co-incubated (at 1:1 and 1:3 cellular ratios) in the setting of *P. aeruginosa* or *S. aureus* infection, but these immune cell types were *not* allowed to come into physical contact. Similar observations were noted as those described above, and thus, data are not shown.

Due to resistin’s ability to effect neutrophil killing of bacteria and its inability to alter monocyte killing, we next examined the ability of both cell types to kill bacteria when co-cultured together in the presence of resistin. This would allow us to determine whether we could mitigate the bacterial killing impairment produced by resistin by altering the ratios of each immune cell type in the mixture. We expected that, by increasing the proportion of neutrophils in co-cultures, we would amplify the inhibitory effects of resistin on bacterial killing. The results depicted in Figs. 3b and 4b indicated that killing of *S. aureus* or *P. aeruginosa* in the presence of resistin was not altered by different ratios of neutrophils to monocytes.

**Fig. 5:**
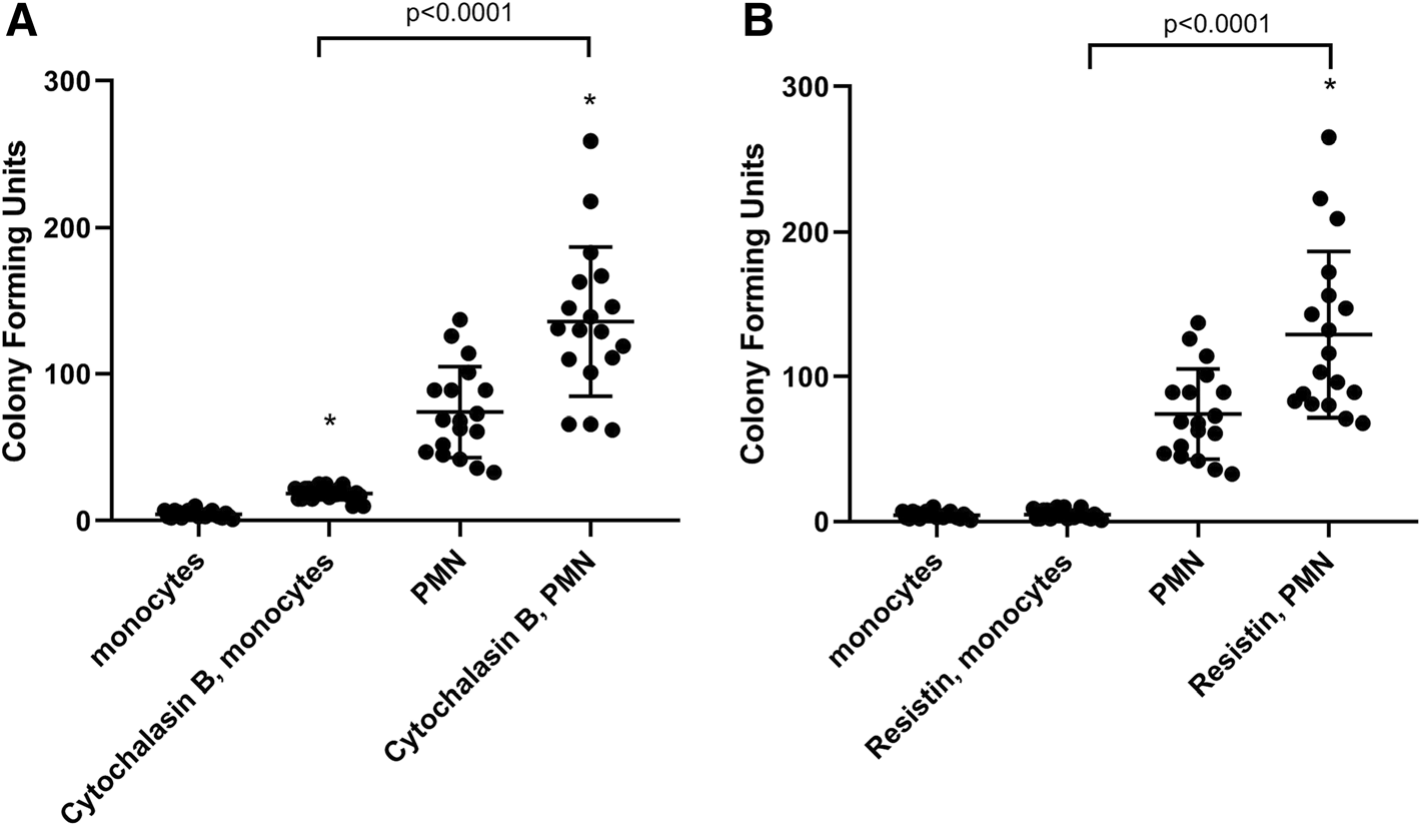
Comparison of the effects of **a** cytochalasin and **b** resistin on the bacterial killing capacity of *P. aeruginosa* by primary human monocytes versus PMN (neutrophils). Cytochalasin significantly impairs bacterial killing by both primary PMN (*P* = 0.0009) and primary monocytes (*P* < 0.0001). Resistin does not affect bacterial killing by monocytes (*P* = 0.91) but significantly impairs killing by neutrophils (*P* = 0.003). *n* = 21 for monocytes and *n* = 18 for neutrophils. Asterisk denotes statistical significance (*P* < 0.05)

**Fig. 6:**
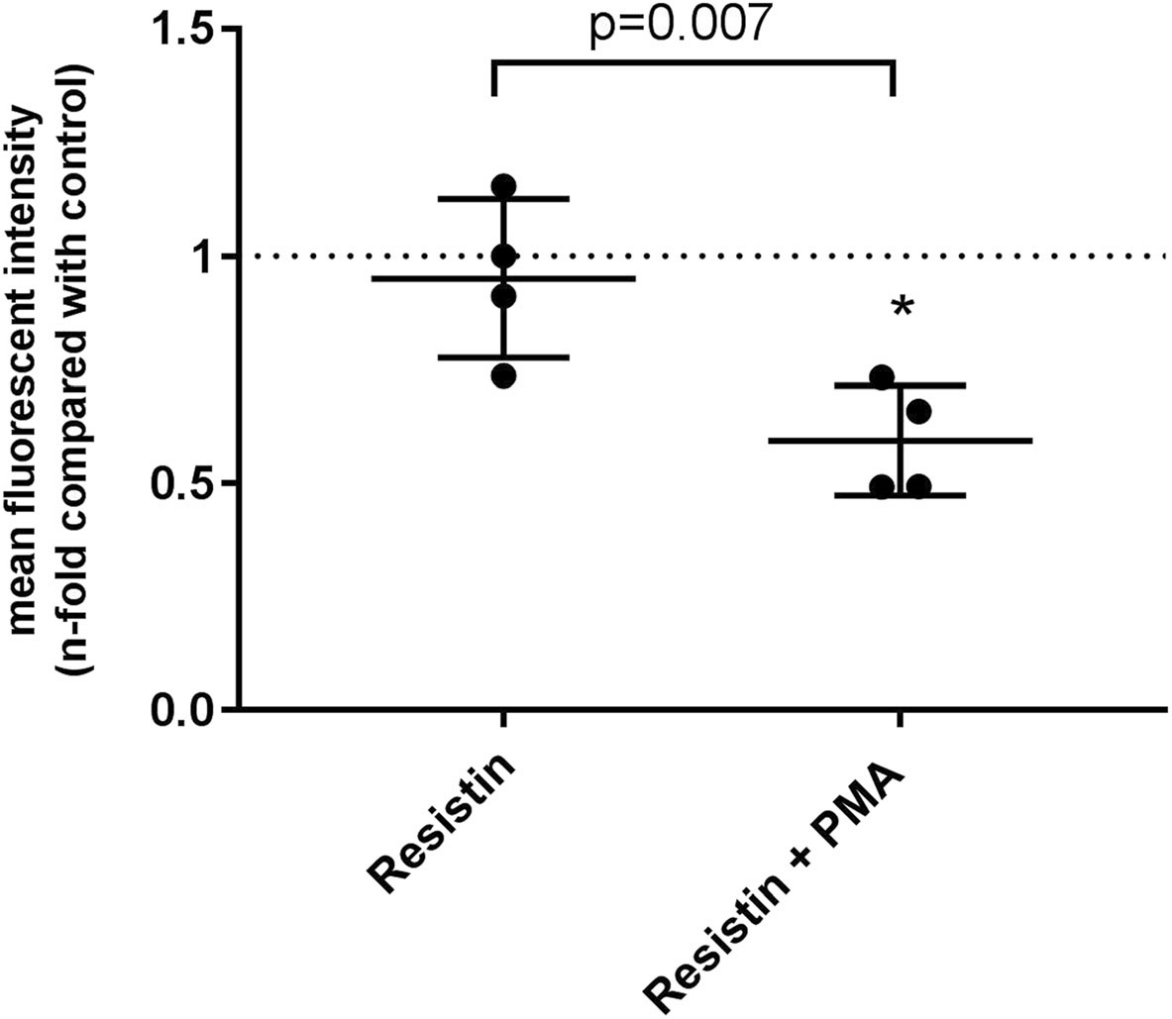
Reactive oxygen species generation in primary neutrophils stimulated resistin in the absence and presence of phorbol 12-myristate 13-acetate (PMA). Asterisk denotes statistical significance (*P* < 0.05) compared to matched control samples (set to 1.0, denoted by the dotted line). Resistin alone does not cause a decrease in reactive oxygen species generation (*P* = 0.84), although addition of the stimulant PMA causes a statistically significant decrease in the ability of neutrophils to generate reactive oxygen species (*P* = 0.003)

In the next set of experiments, we subjected primary neutrophils derived from healthy human volunteers to the same bacterial killing assays described above, in order to confirm the translational relevance of our experimental model. We performed these experiments with *P. aeruginosa* due to the low yield of neutrophils and monocytes obtained from whole blood donated by human volunteers as well as the number of cells required for our standardized assays. The results plotted in Fig. 5 show that resistin exposure inhibits bacterial killing by primary neutrophils (*P* = 0.003) but not by primary monocytes (*P* = 0.91). Quantification of reactive oxygen species generation in the presence and absence of resistin in these primary cells revealed results similar to those previously reported in our neutrophil-differentiated NB4^PMN^ cell lines [15]. Incubation of primary cells with resistin alone did not alter the generation of reactive oxygen species in neutrophils, although incubation with resistin in the presence of the pharmacologic NADPH oxidase stimulator, PMA, significantly decreased reactive oxygen species generation in these cells (Fig. 6).

## Discussion

Immunosuppression is central to sepsis-related morbidity and mortality, and resistin (RETN) appears to be a key mediator within this process [5, 6, 8, 20]. Our current experiments highlight empirically determined responses of innate immune cells to clinically significant, nosocomial infectious agents following resistin exposure. Furthermore, our findings suggest that resistin selectively inhibits bacterial killing of *P. aeruginosa* and *S. aureus* by neutrophils but not by monocytes or macrophages. The exception to this is macrophages, which demonstrate significantly impaired killing of Gram-negative organisms in the presence of resistin. While such an effect could reflect a true physiologic relationship, we cannot exclude the possibility that it represents an artifact of the residual effects of PMA used to differentiate cells, even though PMA was removed from these cells for 24 h prior to experiments.

The direct effect of resistin on neutrophils is clearly detrimental. We previously demonstrated that in these cell types, resistin dose dependently impairs actin polymerization and cellular migration in response to *N*-formylmethionine-leucyl-phenylalanine (fMLP) [9]. Given the critical intracellular role of actin, we surmised that resistin exerts its immunosuppressive effects primarily by impairing intracellular F-actin dynamics. The results from the current series of experiments do not support this proposed mechanism, since monocytes and macrophages are also dependent on F-actin polymerization (as shown by their inhibition with cytochalasin B), yet their ability to eradicate Gram-negative or Gram-positive bacteria was overall unaffected by resistin. Our previously reported [15] and current data have led us to shift our focus away from resistin’s effect on F-actin polymerization and towards a potential direct effect on neutrophil metabolic burst. We have previously demonstrated that reactive oxygen species production is dose dependently impaired by resistin [15]. In the present study, we have further validated our findings in primary neutrophils from human volunteers (Fig. 6).

Our results allow us to generate hypotheses regarding the nature of resistin’s membrane receptor and its restriction to certain leukocyte subsets. There is sparse literature regarding resistin’s receptor, although TLR4 has been proposed as a candidate molecule [21, 22]. TLRs are expressed at higher levels in monocytes compared to neutrophils [19, 23], and resistin also binds with higher affinity to lung monocytes compared with neutrophils [24]. Despite these data, our studies revealed that neutrophils exposed to resistin demonstrated greater impaired killing of Gram-negative organisms compared to an equivalent number of monocytes. Our data thus argue against a physiologically relevant role of TLR4 as a cellular receptor for RETN. However, we cannot rule out the possibility that TLR4 may be only one of several receptors involved in immune cell responses to resistin or that the intracellular TLR4-dependent response to resistin differs between neutrophils and monocytes/macrophages.

Human resistin has been reported to be detrimental in helminth infection as it promotes the production of pro-inflammatory cytokines while impairing monocyte migration and monocyte-driven parasite killing [24]. However, in a transgenic mouse model of endotoxemia, Jang et al. subsequently proposed that this cytokine inhibits the interaction between LPS and TLR4 at the cell surface of immune cells, thus improving survival following LPS exposure [22]. A limitation of the latter study is the use of transgenic mice expressing recombinant human resistin, a model which may introduce confounding factors that are difficult to control experimentally. This same study also utilized LPS to simulate Gram-negative infection, an approach having inherent limitations when modeling human sepsis [25, 26]. Our direct incubation of immune cells with opsonized bacteria did not replicate the protective effects of resistin demonstrated in the aforementioned model of endotoxemia. While the conflicting findings may emphasize the context dependence of resistin’s cellular effects, our results suggest that resistin’s direct effect is, at worst, harmful to neutrophils both in the context of Gram-positive and Gram-negative infection and, at best, neutral to monocytes or macrophages.

Prior reports have indicated that monocytes respond to infection by synthesizing cytokines and that these cytokines, in turn, affect the survival and proliferation of neutrophils [27]. Additionally, neutrophil responses to bacterial invasion (including cell survival and expression of adhesion molecules in response to LPS) are amplified in the presence of monocytes [19]. We hypothesized that this synergistic killing effect might be able to mitigate resistin’s effects in vitro, and we designed a cell-based model to test this hypothesis. We co-incubated monocytes and neutrophils, but we did not achieve an improvement in the neutrophil bacterial killing of Gram-positive or Gram-negative bacteria, even when high concentrations of monocytes were used. The absence of a synergistic effect was still evident in cells incubated with resistin. While no effect may indeed exist, these data may be a result of an oversimplified experimental model which may not reflect in vivo conditions during an infection. Alternatively, the timing of our incubations may not have been reflective of the kinetics of “co-incubation” in vivo. Monocytes, on the other hand, demonstrated superior killing of *S. aureus* when compared with neutrophils, while they exhibited diminished killing when co-incubated with neutrophils at preserved total cellular ratios. This observation may be explained by the presence of neutrophils preventing physical contact between monocytes and bacteria or by impaired monocyte function caused by residual retinol utilized to differentiate NB4^PMN^ cells.

One strength of our study is the simplicity of our experimental design. We modeled cell-based responses to direct bacterial exposure, with minimal pre-exposure cellular or antigen processing. Unlike other studies, we did not use LPS to emulate Gram-negative infection, neither did we focus specifically on cell surface receptors posited to be involved in Gram-negative or Gram-positive infection. Furthermore, in contrast to earlier studies suggesting TLR4 as a receptor for resistin [21], we utilized concentrations of resistin that are reflective of those present in vivo during sepsis and kidney disease [15], in order to prevent artificial receptor activation by supraphysiologic stimulant concentrations.

While our approach lacks the complexity of receptor-targeted studies or transgenic rodent models, the medical literature is still ambiguous regarding the fundamental immune cell-based response to resistin following an acute infection [13]. Here, we offer further evidence that neutrophils exposed to human resistin exhibit impaired bacterial killing, a characteristic feature of sepsis-induced immunosuppression. These data are consistent with the hypothesis that resistin selectively causes neutrophil dysfunction during sepsis/septic shock. Combined with our previous findings relating to reduced neutrophil migration and reduced production of reactive oxygen species with hyperresistinemia [9, 15], we maintain that resistin alone can replicate the immunosuppressed phenotype demonstrated in these disease states.

## Conclusions

Human resistin significantly impairs neutrophil killing of Gram-positive and Gram-negative organisms, while it does not directly affect the ability of monocytes and macrophages to kill these organisms. Increasing the proportion of monocytes in neutrophil/monocyte co-cultures does not mitigate the negative effects of resistin on bacterial killing by neutrophils.

## Data Availability

All data generated or analyzed during this study are included in this published article.

## Abbreviations

AKI: Acute kidney injury
ATRA: All-trans retinoic acid
FBS: Fetal bovine serum
fMLP: *N*-Formylmethionine-leucyl-phenylalanine
LPS: Lipopolysaccharide
PMA: Phorbol 12-myristate 13-acetate
PMN: Polymorphonuclear neutrophils
RETN: Resistin
ROS: Reactive oxygen species
TLR: Toll-like receptor

## References

[1] Hotchkiss RS, Monneret G, Payen D (2013). Immunosuppression in sepsis: a novel understanding of the disorder and a new therapeutic approach. Lancet Infect Dis.

[2] Boomer JS (2011). Immunosuppression in patients who die of sepsis and multiple organ failure. JAMA.

[3] Bonavia A (2018). Clinical utility of extracorporeal cytokine hemoadsorption therapy: a literature review. Blood Purif.

[4] Cohen G (2008). Resistin inhibits essential functions of polymorphonuclear leukocytes. J Immunol.

[5] Macdonald SP (2014). Sustained elevation of resistin, NGAL and IL-8 are associated with severe sepsis/septic shock in the emergency department. PLoS One.

[6] Koch A (2009). Serum resistin levels in critically ill patients are associated with inflammation, organ dysfunction and metabolism and may predict survival of non-septic patients. Crit Care.

[7] Johansson L (2009). Neutrophil-derived hyperresistinemia in severe acute streptococcal infections. J Immunol.

[8] Sunden-Cullberg J (2007). Pronounced elevation of resistin correlates with severity of disease in severe sepsis and septic shock. Crit Care Med.

[9] Singbartl K (2016). Reversal of acute kidney injury-induced neutrophil dysfunction: a critical role for resistin. Crit Care Med.

[10] Hillenbrand A (2010). Sepsis induced changes of adipokines and cytokines - septic patients compared to morbidly obese patients. BMC Surg.

[11] Kellum JA (2016). The effects of alternative resuscitation strategies on acute kidney injury in patients with septic shock. Am J Respir Crit Care Med.

[12] Nüsken KD (2006). Circulating resistin concentrations in children depend on renal function. Nephrol Dial Transplant.

[13] Pine GM, Batugedara HM, Nair MG (2018). Here, there and everywhere: resistin-like molecules in infection, inflammation, and metabolic disorders. Cytokine.

[14] Bonavia Anthony, Singbartl Kai (2017). A review of the role of immune cells in acute kidney injury. Pediatric Nephrology.

[15] Bonavia A (2017). Hemoadsorption corrects hyperresistinemia and restores anti-bacterial neutrophil function. Intensive Care Med Exp.

[16] Tsuchiya S (1980). Establishment and characterization of a human acute monocytic leukemia cell line (THP-1). Int J Cancer.

[17] Stokes RW, Doxsee D (1999). The receptor-mediated uptake, survival, replication, and drug sensitivity of Mycobacterium tuberculosis within the macrophage-like cell line THP-1: a comparison with human monocyte-derived macrophages. Cell Immunol.

[18] Hampton MB, Vissers MC, Winterbourn CC (1994). A single assay for measuring the rates of phagocytosis and bacterial killing by neutrophils. J Leukoc Biol.

[19] Sabroe I (2002). Toll-like receptor (TLR)2 and TLR4 in human peripheral blood granulocytes: a critical role for monocytes in leukocyte lipopolysaccharide responses. J Immunol.

[20] Vassiliadi DA (2012). Serial changes in adiponectin and resistin in critically ill patients with sepsis: associations with sepsis phase, severity, and circulating cytokine levels. J Crit Care.

[21] Tarkowski A (2010). Resistin competes with lipopolysaccharide for binding to toll-like receptor 4. J Cell Mol Med.

[22] Jang JC (2017). Human resistin protects against endotoxic shock by blocking LPS-TLR4 interaction. Proc Natl Acad Sci U S A.

[23] Dale DC, Boxer L, Liles WC (2008). The phagocytes: neutrophils and monocytes. Blood.

[24] Jang JC (2015). Macrophage-derived human resistin is induced in multiple helminth infections and promotes inflammatory monocytes and increased parasite burden. PLoS Pathog.

[25] Lewis AJ, Seymour CW, Rosengart MR (2016). Current murine models of sepsis. Surg Infect.

[26] Seok J (2013). Genomic responses in mouse models poorly mimic human inflammatory diseases. Proc Natl Acad Sci U S A.

[27] Suzuki T (2000). Comprehensive gene expression profile of LPS-stimulated human monocytes by SAGE. Blood.

